# Fusion of photon-counting CT and MRI improves assessment of the knee: a pilot study

**DOI:** 10.1016/j.ostima.2026.100470

**Published:** 2026-07-16

**Authors:** Zhaoye Zhou, Theresa Sophie Patzer, Mohamad Alzaher, Ali Guermazi, Dorra Guermazi, Karim Sariahmed, Allyson Zheng, Marco L. Loggia, Mohamed Jarraya, Fang Liu

**Affiliations:** a Athinoula A. Martinos Center for Biomedical Imaging, Harvard Medical School, Boston, MA, United States; b Department of Radiology, Massachusetts General Hospital, Boston, MA, United States; c Department of Diagnostic and Interventional Radiology, University Hospital Würzburg, Würzburg, Germany; d Quantitative Imaging Center, Department of Radiology, Boston University School of Medicine, Boston, MA, United States; e Department of Radiology, VA Boston Healthcare System, West Roxbury, MA, United States; f Warren Alpert Medical School of Brown University, Providence, RI, United States; g Section of General Internal Medicine, Department of Medicine, Boston Medical Center, Boston, MA, United States

**Keywords:** Osteoarthritis, Knee, Cartilage, MRI, Photon counting CT

## Abstract

**Purpose:**

The complementary information gained by combining photon counting CT (PCCT) and MRI may enhance the evaluation of the osteochondral unit. Our aim is to evaluate whether fusing PCCT and MRI improves diagnostic clarity and segmentation consistency for osteochondral lesions compared with MRI alone.

**Materials and methods:**

24 participants (15 with knee OA and 9 healthy controls) underwent PCCT and 3T MRI between October 2024 and May 2025. Images were co-registered and fused using a mutual information-based algorithm. Two musculoskeletal radiologists rated diagnostic clarity on 5-point Likert scales. Inter-reader agreement was quantified with the intraclass correlation coefficient (ICC) and Cohen kappa. Another musculoskeletal radiologist and a radiology trainee performed manual segmentations of bones, cartilage, meniscus, and osteophytes on PCCT-MRI fusion or MRI alone. Inter- and intra-rater segmentation consistency was evaluated using Dice similarity coefficient and surface Dice (sDice). Paired comparisons were analyzed with the Wilcoxon signed-rank test.

**Results:**

Fusion imaging yielded significantly higher clarity scores for cartilage thickness (3.91 vs 4.95), articular surface (3.93 vs 4.63), and osteophyte boundaries (4.00 vs 4.83; all *p* < .05). Inter-reader agreement improved (ICC, from 0.43 to 0.88; Cohen kappa, from 0.42 to 0.89). Quantitative analysis showed greater inter- and intra-rater consistency for cartilage (Dice, 0.70 vs 0.81; sDice, 0.66 vs 0.78) and bone (Dice, 0.93 vs 0.95; sDice, 0.61 vs 0.86; all *p* < .01).

**Conclusion:**

PCCT-MRI fusion enhances diagnostic clarity and inter-reader agreement. Fusion also increases segmentation consistency for cartilage and bone, supporting its potential clinical value for comprehensive osteochondral evaluation.

## 1 Introduction

The cartilage and subchondral bone form a functional unit whose integrity is essential for joint health and whose disruption contributes to the pathogenesis of osteoarthritis (OA) [1]. Alterations of the subchondral bone (bone plate and trabecular bone) and bone marrow lesions are not merely changes secondary to cartilage loss but can contribute to OA progression and symptoms [2,3]. MRI is particularly valuable for evaluating soft tissues in the joint, including cartilage, menisci, and bone marrow lesions [4]. However, its susceptibility to chemical shift artifacts [5,6] at the interface between the subchondral bone plate and the fat-containing medullary cavity often leads to an overestimation of the subchondral bone plate and misinterpretation of cartilage lesion depth and cartilage thickness [7,8]. Compared with conventional MRI, ultrashort echo time and zero echo time techniques substantially improve visualization of short-T2 tissues within the knee by capturing rapidly decaying signals that are largely undetectable with standard clinical sequences [[9], [10], [11], [12], [13], [14], [15], [16]]. These techniques can further depict the osteochondral junction as a distinct signal band at the cartilage-bone interface, enabling more detailed assessment of osteochondral anatomy and early degenerative changes. While these techniques significantly improve interface visualization, pure MRI approaches still face challenges in spatial resolution for detecting the subchondral bone plate and changes in the subchondral trabecular bone [17]. Photon-counting computed tomography (PCCT) is an emerging CT technology that offers the major advantage of increased spatial resolution compared to energy-integrating detector CT systems, with the additional benefit of improved dose efficiency, with an estimated 30-50% dose reduction [18,19]. These characteristics make PCCT a promising clinical research tool for evaluating subchondral bone morphology in OA [20].

CT-MRI fusion has previously been explored in the knee to improve anatomic co-localization for automated preoperative planning in ACL reconstruction [21]. However, PCCT offers new opportunities for high-resolution imaging of bone, which could be leveraged to improve our understanding of the relationship between trabecular bone microarchitecture and MRI-visible osteoarthritis features, including cartilage abnormalities, bone marrow lesions, synovitis, and other soft-tissue changes. PCCT offers superior spatial resolution and enhanced visualization of cortical and subchondral bone structures, compared to traditional energy-integrating detector CT, while MRI offers excellent soft tissue contrast. By integrating these capabilities, PCCT-MRI fusion could generate a comprehensive depiction of osteochondral and intra-articular structures within a fused modality, addressing the limitations inherent to each modality alone. This approach may be particularly valuable for evaluating changes in the subchondral bone plate and trabecular microarchitecture in the vicinity of cartilage damage.

Despite these intriguing possibilities, the diagnostic value of PCCT-MRI fusion for knee assessment has not been systematically validated. Therefore, the purpose of this study was to evaluate the hypothesis that PCCT-MRI fusion provides superior diagnostic utility compared with MRI alone, by assessing both qualitative improvements in diagnostic clarity for the radiologist and quantitative gains in segmentation consistency of osteochondral structures.

## 2 Materials and methods

We designed a systematic study workflow comprising patient cohort construction, image acquisition, image registration and fusion, radiologist qualitative assessment, quantitative segmentation evaluation, and statistical analysis. Each step of the workflow is described in detail below.

### 2.1 Patient cohort construction

In this study, we used data from the DIAMOND knee observational study that aims to identify baseline imaging and pain characteristics predictive of chronic postoperative pain after total knee replacement. In the current study we analyzed data from the participants who were scanned between October 2024 and May 2025. Patient-participants received concurrent MRI and PCCT evaluations of both knees (participants with a prior contralateral knee replacement had only one knee examined). The evaluations were part of a comparative study of patients with advanced knee OA and asymptomatic patients, which aimed to identify structural correlations to pain mechanisms in knee OA. This study was approved by the Institutional Review Board at Mass General Brigham), and written informed consent was obtained from all participants prior to inclusion.

### 2.2 Study eligibility for the parent DIAMOND knee study

#### 2.2.1 Patients

Inclusion criteria included being 40 years or older, sufficient English proficiency to complete study procedures, a diagnosis of knee OA in one or both knees, and a scheduled total knee arthroplasty. Exclusion criteria included inability to understand study instructions or scales, contraindications to MRI scanning, a current illicit substance use disorder, intra-articular steroid injections or prolonged systemic corticosteroids within two months of scanning, and current or recent medical conditions that may affect neuroinflammation, such as HIV, rheumatoid arthritis, or lupus. Participants were also excluded because of poor image quality or poor registration due to substantial differences in image modalities and resolution between PCCT and MRI.

#### 2.2.2 Healthy Controls

Inclusion and exclusion criteria were the same for both groups, although healthy controls had additional exclusions for knee osteoarthritis or other chronic pain conditions and depression or anxiety requiring treatment.

### 2.3 Image acquisition

All PCCT and MRI examinations were performed at [our institution].

PCCT scans were performed using a NAEOTOM Alpha PCCT scanner (Siemens Healthineers, Forchheim, Germany) with the patient in a supine position and the knee joint fully extended. The acquisition parameters included a tube voltage of 120 kVp with dose modulation strategy (Image Quality level 75), resulting in a CT dose index volume of approximately 7-10 mGy and an effective mAs of 90-100. Scans were acquired using the Ultra High Resolution (UHR) mode with a rotation time of 0.5 seconds, a pitch of 0.85, a slice thickness of 0.2 mm, and a slice increment of 0.2 mm. Images were reconstructed using a sharp bone kernel (Br89) with a matrix size of 1024 × 1024 and a field of view ranging from 200 mm to 400 mm. The resulting voxel size was variable, ranging from 0.2 mm to 0.4 mm, depending on the field of view and matrix.

MRI examinations were performed on a Siemens Healthineers, MAGNETOM Prisma 3T system with a 15-channel knee coil. Each patient underwent multiplanar imaging, including axial, coronal, and sagittal planes. The protocol consisted of sagittal proton density-weighted fat-suppressed, axial and coronal T2-weighted fat-suppressed (T2-FS), and coronal T1-weighted sequences. Our protocol combines both proton density-weighted and T2-weighted sequences with fat suppression to balance high sensitivity and specificity for detection in water signal, respectively [22]. We used a slice thickness of 4.4 mm and an in-plane resolution of 0.42 mm × 0.42 mm. For subsequent registration, fusion, and segmentation analyses, the coronal T2-FS sequence was selected because it provides high sensitivity for fluid and edema, clear delineation of articular cartilage and meniscal morphology and complements the osseous detail from PCCT, thereby maximizing the complementary information available in a fused modality.

### 2.4 Image registration & fusion

Image registration was performed to correct patient positioning differences, physiological motion, and variations in knee flexion that can occur between the two acquisitions. For each PCCT/MRI pair, MRI served as the fixed reference and PCCT as the moving image because MRI offers superior soft-tissue contrast and a standardized, stable knee position for alignment. A rigid registration was initially performed using a mutual information-based algorithm [23] implemented with elastix [24]. Rigid registration was performed using Advanced Mattes mutual information with an Euler transform (64 bins, 3 multi-resolution levels, 500 iterations). After this, a non-rigid B-spline registration was applied using Mattes mutual information (60 bins, weight = 1), a bending-energy penalty (weight = 50), a final control-point grid spacing of 15 mm, 3 resolution levels, and up to 5000 ASGD iterations. Registration accuracy was independently evaluated by two musculoskeletal radiologists through visual assessment of the alignment of major anatomical landmarks, including the femoral condyles, tibial plateau, tibial spines, and cortical bone contours.

After registration, PCCT and MRI volumes were fused using a multi-step process ensuring anatomical accuracy and quantitative integrity. Because their intensity scales differ (absolute Hounsfield Units (HU), vs arbitrary MRI units), normalization was applied to harmonize both modalities. Specifically, MRI signal intensities were scaled to a 0-1 range using min-max normalization within each volume:

(1) $$I_{norm}^{mri}=\frac{I^{mri}-I_{min}^{mri}}{I_{max}^{mri}-I_{min}^{mri}},$$

where $I_{min}$ and $I_{max}$ represent the minimum and maximum intensity values within each MRI volume.

For PCCT volumes, the original HU values were windowed using clinically relevant settings (e.g., window center (WC) and window width (WW)) for bone (WC: 350-500 HU; WW: 1500-2000 HU) and soft tissue (WC: 40 HU; WW: 400 HU). The windowed intensity was calculated as:

(2) $$I_{windowed}^{PCCT}=\min\left(\left(\max(I^{PCCT},L\right),U\right),$$

where $L=\text{WC}-\frac{\text{WW}}{2}$ and $U=\text{WC}+\frac{\text{WW}}{2}$.

The windowed values were then rescaled to a normalized 0-1 range using min-max scaling:

(3) $$I_{norm}^{PCCT}=\;\frac{I_{windowed}^{PCCT}-L}{U-L}$$

The fusion process was executed within the 3D Slicer platform using a deterministic weighted blending algorithm to combine the normalized PCCT and MRI volumes [25]. To optimize visualization of both fine osseous structures and soft tissue features, a 50:50 blend ratio was empirically determined by two senior radiologists. The algorithm in 3D Slicer platform yields consistent, reproducible fusion outputs across repeated runs when given identical inputs from the previous registration steps.

### 2.5 Qualitative assessment

The qualitative assessment aimed to determine whether PCCT-MRI fusion improves anatomical and pathological visualization over MRI alone and yields more consistent interpretations across readers. Independent diagnostic review was performed by two fellowship-trained musculoskeletal radiologists (Reviewer A and Reviewer B, with 30 and 15 years of experience, respectively). All reviewers were blinded to patient clinical information and to the imaging modality during scoring. MRI-only, and PCCT-MRI fusion cases were presented in a randomized order to each reader to minimize recall bias and potential preference effects.

For each case, reviewers evaluated the clarity of key anatomical structures and pathological features using a standardized 5-point Likert scale (1 = very poor, 5 = excellent). The categories evaluated included (1) sharpness of the subchondral bone plate and overall cartilage thickness; (2) sharpness of cartilage surface; (3) diagnostic confidence of meniscal tear morphology; (4) sharpness of osteophyte boundaries. These criteria were selected because clear delineation of subchondral bone plate and cartilage surface is crucial for accurate grading of cartilage defects and subchondral bone pathology in OA [[26], [27], [28], [29], [30]].

### 2.6 Quantitative segmentation

A quantitative segmentation analysis was performed to evaluate inter- and intra-rater consistency. Another musculoskeletal radiologist and a radiology trainee (Reviewer C and Reviewer D, with 5 and 2 years of experience, respectively) independently performed manual segmentations on both PCCT-MRI fusion and MRI-only cases on the slice providing the most diagnostic information. The slice was jointly selected by Reviewers C and D, and confirmed by Reviewer B. Specifically, the coronal slice showing the maximal extension of the tibial spines was selected for each patient dataset. Utilizing the tibial spines as a standardized landmark ensured high structural consistency and reproducibility across the cohort. Moreover, it yields the optimal visualization of the meniscal body, cartilage, the cruciate ligaments and bone, thereby aligning the image selection directly with our clinical target for precise joint space and soft-tissue boundary assessment. The segmented structures included bone (femur and tibia, separately), articular cartilages (femoral and tibial), menisci (medial and lateral), and osteophytes. To minimize potential learning effects and bias, the order of cases and modalities was randomized, and all annotations were performed using the same version of 3D Slicer under identical display settings. Reviewers were blinded to each other’s results during annotation.

Inter-rater reliability was assessed by comparing segmentations from Reviewers C and D, while intra-rater consistency was evaluated after each reviewer repeated the task two weeks later. Dice similarity coefficient [31] and surface Dice (sDice, τ = 1 mm) [32] quantified segmentation overlap and boundary agreement for each modality.

### 2.7 Statistical analysis

For the qualitative assessment, clarity scores were summarized as mean ± standard deviation (SD). Paired comparisons between MRI-only and PCCT-MRI fusion images were performed using the Wilcoxon signed-rank test [33]. Inter-reader agreement was evaluated for each modality using the intraclass correlation coefficient (ICC) [34] and Cohen kappa [35] to provide a comprehensive, dual-perspective assessment of agreement. Cohen kappa was applied to evaluate chance-corrected agreement for the ordinal classifications, while the ICC was used by treating the Likert scores as quasi-continuous variables to quantify absolute scoring consistency across readers.

For quantitative segmentation agreement, Dice [36] and sDice [37] were calculated for each structure and reported as mean ± SD. Differences between modalities were assessed with the Wilcoxon signed-rank test. All statistical tests were two-tailed, and a p-value < 0.05 was considered statistically significant.

All analyses were performed in Python (v3.12). The Pingouin package was used to compute ICC and Cohen kappa, SciPy for nonparametric statistical testing, and Plotly for data visualization.

## 3 Results

### 3.1 Sample characteristics

**Fig. 1:**
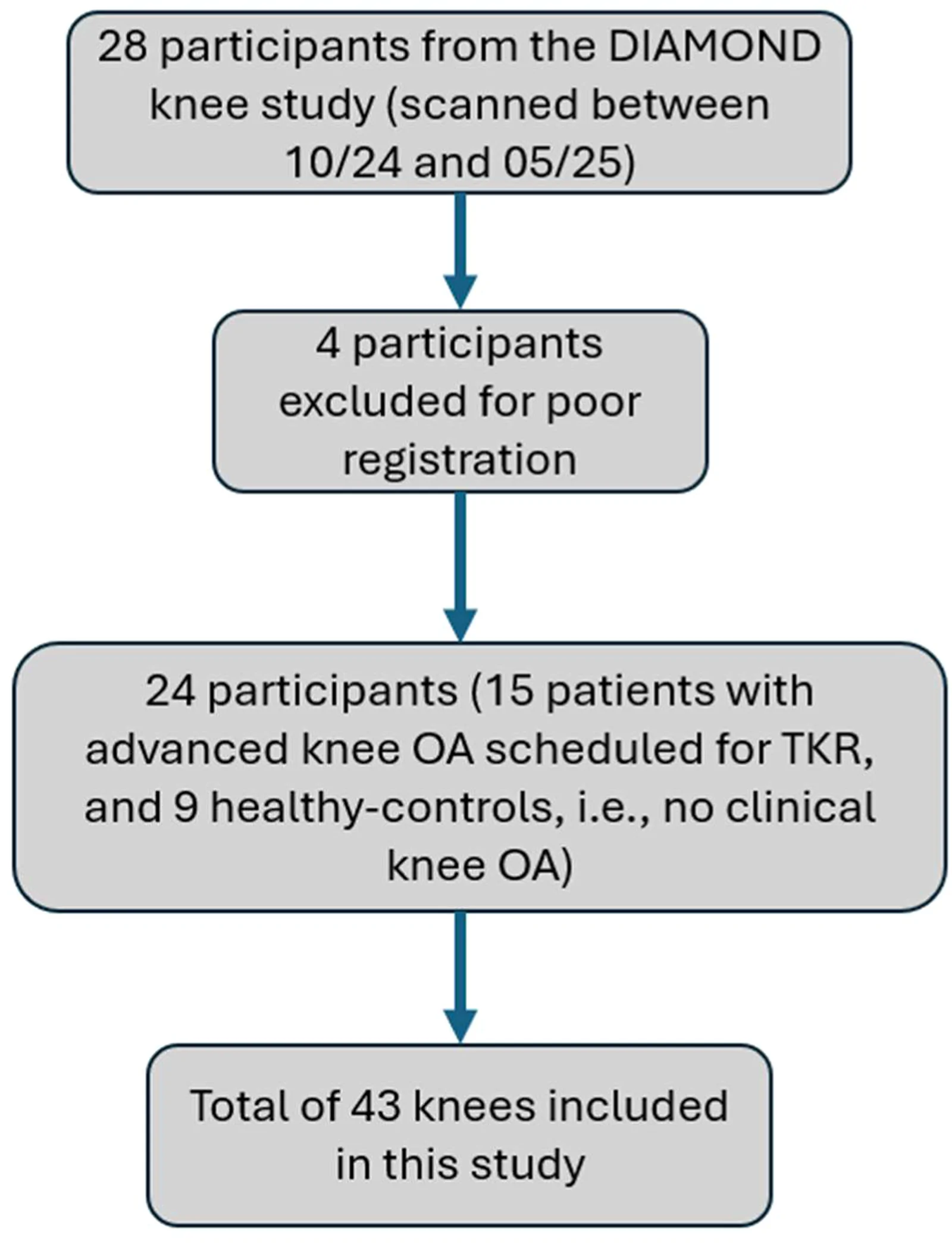
Flowchart of sample selection.

A total of 28 participants were initially identified for both MRI and PCCT examinations. Of these, 4 were excluded due to poor registration caused by positional misalignments or patient motion, 24 patients (15 with advanced knee OA and 9 healthy controls) who contributed 43 knees for the current analysis (Fig. 1). The study sample was 50% female, the median age was 67 years (IQR: 46-81 years), and the mean BMI was 26.8 kg/m² (SD ± 4.6). Kellgren-Lawrence (KL) grades were distributed as follows: KL 0, 5 patients (21%); KL 1, 4 (17%); KL 2, 6 (25%); KL 3, 6 (25%); and KL 4, 3 (12%). The mean interval between PCCT and MRI was 3.4 ± 9.7 days (Table 1).

**Table 1:** Summary of demographic and clinical characteristics of the study cohort (n = 24). Values are presented as mean ± standard deviation (SD) with ranges in parentheses or as counts with percentages in parentheses. KOA = knee osteoarthritis; KL = Kellgren-Lawrence.

Characteristic	Value
**Number of Subjects**	24
**Diagnostic group, n (%)**	KOA: 15 (62.5%) Healthy controls: 9 (37.5%)
**Age, mean ± SD (range), years**	65.0 ± 9.9 (46-81)
**Sex, n (%)**	Male: 12 (50%) Female: 12 (50%)
**BMI, mean ± SD (range), kg/m²**	26.8 ± 4.6 (17.92-36.31)
**KL grade**	KL 0: 5 (21%) KL 1: 4 (17%) KL 2: 6 (25%) KL 3: 6 (25%) KL 4: 3 (12%)
**Time interval between PCCT and MRI, mean ± SD (days)**	3.4 ± 9.7

### 3.2 Qualitative assessment results

**Fig. 2:**
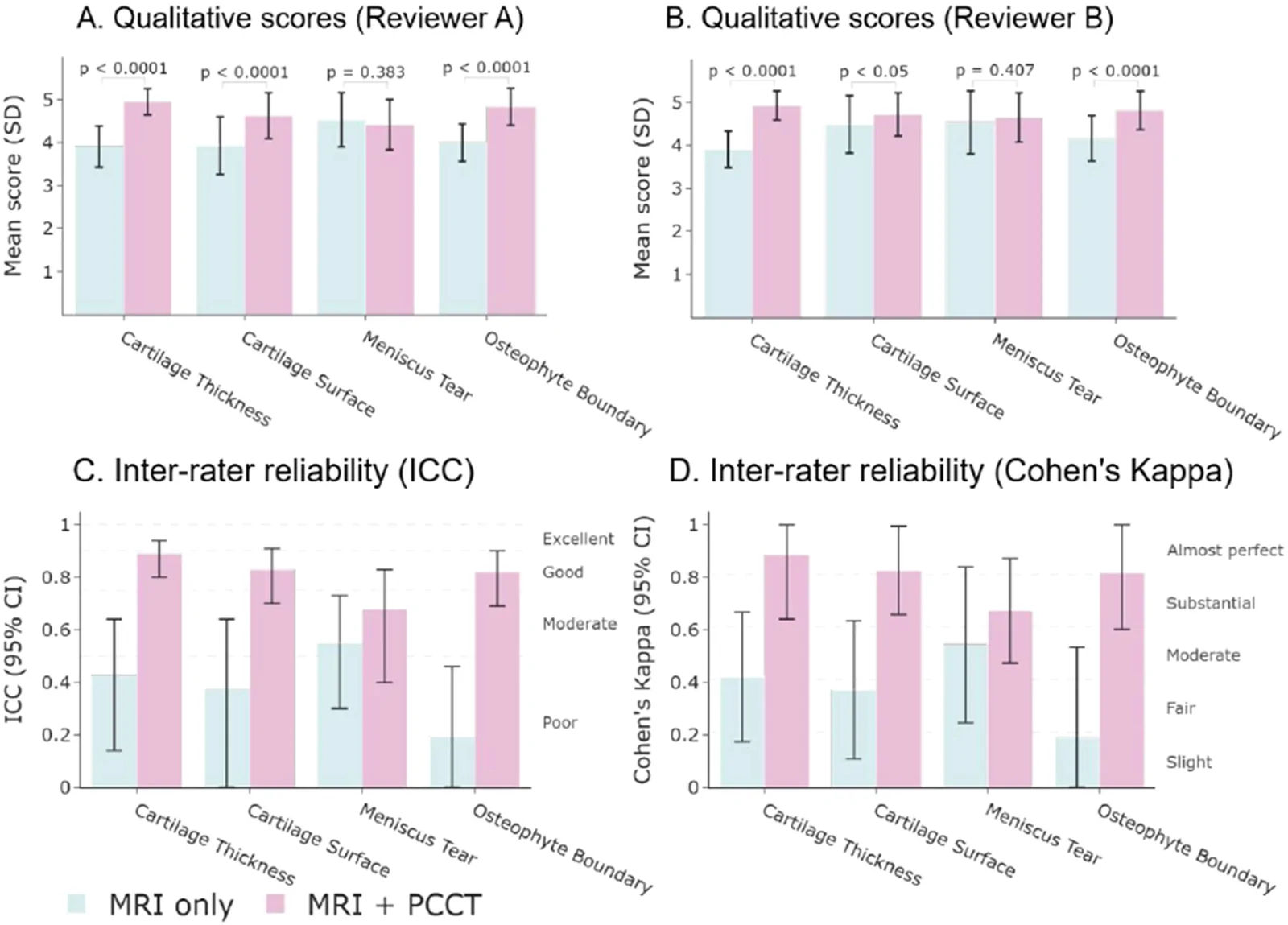
Radiologist qualitative assessment results Comparing MRI alone and PCCT-MRI fusion. (A-B) Mean clarity scores (± SD) for MRI alone and PCCT-MRI fusion images evaluated by two musculoskeletal radiologists (Reviewer A and Reviewer B). (C) Inter-rater reliability measured by intraclass correlation coefficient (ICC) with 95% confidence intervals (CI). (D) Inter-rater reliability measured by Cohen kappa with 95% CI.

Fig. 2 and Table 2 summarize the qualitative diagnostic review performed by the two musculoskeletal radiologists (Reviewer A and B). Both readers consistently reported significantly higher clarity scores with PCCT-MRI fusion compared with MRI alone. Specifically, for the sharpness of cartilage thickness, mean scores increased from 3.91 ± 0.48 for MRI to 4.95 ± 0.30 forfusion for Reviewer A and from 3.91 ± 0.43 to 4.93 ± 0.35 for Reviewer B (both *p* < 0.0001). For the sharpness of cartilage surface, Reviewer A’s mean score improved from 3.93 ± 0.67 to 4.63 ± 0.54 (*p* < 0.0001), while Reviewer B’s score increased from 4.49 ± 0.67 to 4.72 ± 0.50 (*p* < 0.05). Similarly, osteophyte boundary delineation scores improved significantly, rising from 4.00 ± 0.44 (MRI) to 4.83 ± 0.43 (fusion) for Reviewer A and from 4.16 ± 0.53 to 4.81 ± 0.45 for Reviewer B (both *p* < 0.0001). For meniscal tear evaluation, scores were comparable between fusion and MRI-only images, with no statistically significant differences (Reviewer A: 4.54 ± 0.63 vs 4.42 ± 0.59, p = 0.383; Reviewer B: 4.53 ± 0.74 vs 4.65 ± 0.57, p = 0.407). In addition, inter-reader agreement was also enhanced with fusion images. For example, the ICC for cartilage thickness increased from 0.43 [95% CI: 0.14-0.64] with MRI to 0.88 [95% CI: 0.80-0.94] with fusion, and Cohen kappa similarly improved from 0.42 [95% CI: 0.17-0.67] to 0.89 [95% CI: 0.64-1.00].

**Table 2:** Radiologist qualitative assessment results. Mean clarity scores (± SD) from two musculoskeletal radiologists for MRI alone and PCCT-MRI fusion images across key diagnostic categories. Inter-reader agreement is reported using intraclass correlation coefficient (ICC) and Cohen kappa with 95% confidence intervals (CI).

Items	Reviewer A (mean ± SD)		Reviewer B (mean ± SD)		ICC (mean [95% CI])		Cohen kappa (mean [95% CI])	
	MRI	Fusion	MRI	Fusion	MRI	Fusion	MRI	Fusion
**Cartilage Thickness**	3.91±0.48	4.95±0.30*	3.91±0.43	4.93±0.35*	0.43 [0.14, 0.64]	0.88 [0.80, 0.94]	0.42 [0.17, 0.67]	0.89 [0.64, 1.00]
**Cartilage Surface**	3.93±0.67	4.63±0.54*	4.49±0.67	4.72±0.50*	0.38 [0.00, 0.64]	0.83 [0.70, 0.91]	0.37 [0.11, 0.63]	0.83 [0.66, 1.00]
**Meniscus Tear**	4.54±0.63	4.42±0.59	4.53±0.74	4.65±0.57	0.55 [0.30, 0.73]	0.68 [0.40, 0.83]	0.54 [0.25, 0.84]	0.67 [0.47, 0.87]
**Osteophyte Boundary**	4.00±0.44	4.83±0.43*	4.16±0.53	4.81±0.45*	0.19 [0.00, 0.46]	0.82 [0.69, 0.90]	0.19 [0, 0.53]	0.81 [0.60, 1.00]

*Significance levels:* *p < 0.05.

**Fig. 3:**
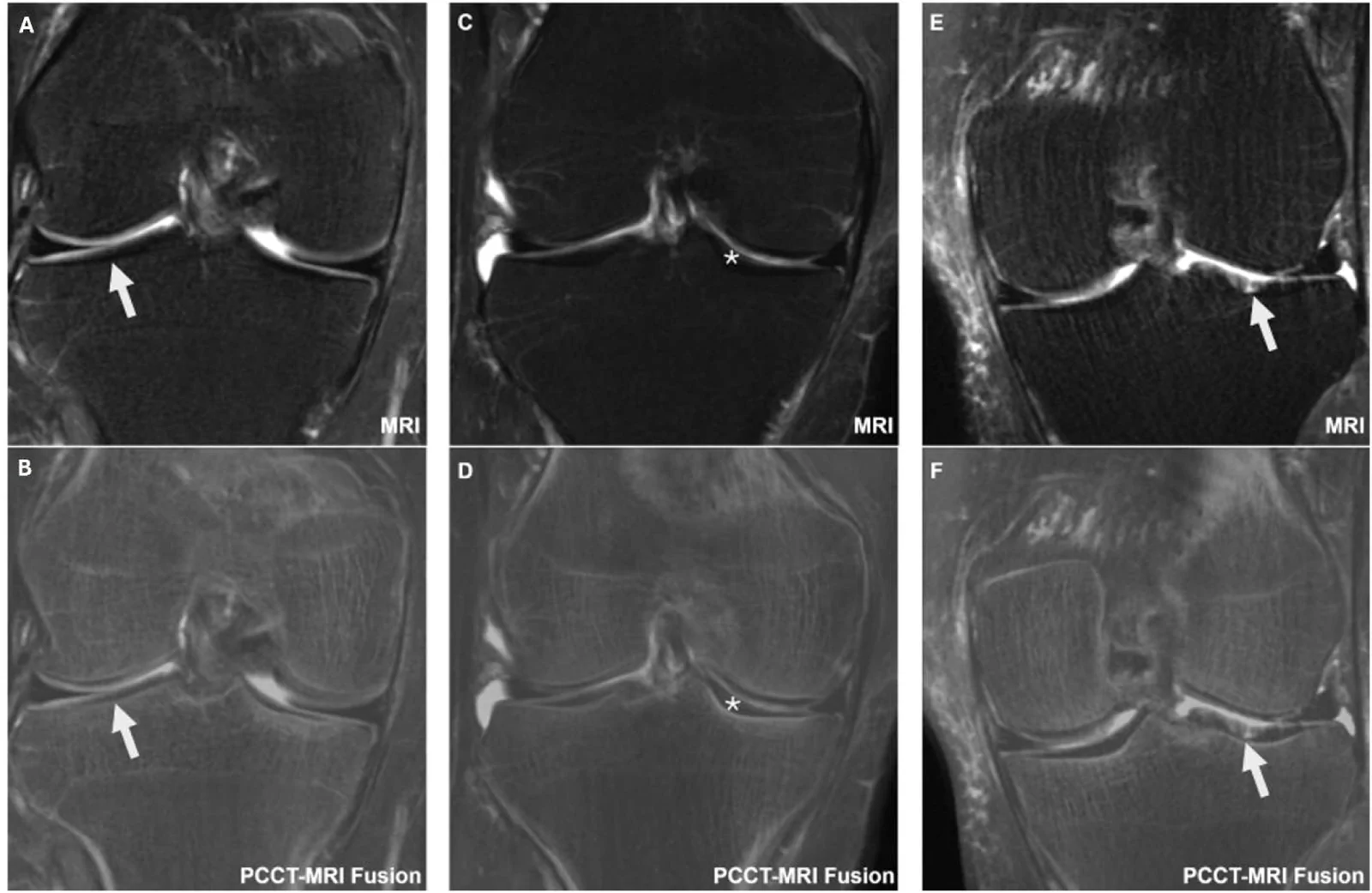
Representative examples comparing MRI alone (upper row) and PCCT-MRI fusion (lower row) images. (A) Coronal T2-weighted MRI with fat-suppression with normal tibial and femoral cartilage thickness and surface. (B) The PCCT-MRI fusion image shows a much better delineation of the subchondral bone plate (arrow) compared to the MRI alone. (C) Coronal T2-weighted MRI with fat-suppression showing thinning of the tibial and femoral cartilage. The precise thickness of the femoral compartment cartilage is difficult to assess given the low T2 signal of the deep cartilage layer. (D) The PCCT-MRI fusion image shows a much clearer delineation of the subchondral bone plate (star) allowing a more accurate assessment of the degree of cartilage loss. (E) Coronal T2-weighted MR image with fat suppression showing focal high-grade cartilage loss of the medial tibial plateau (arrow). (F) The combined image shows a focal irregularity of the underlying subchondral bone plate with a remaining layer of deep cartilage.

Examples comparing visualization on MRI alone and PCCT-MRI fusion are shown in Fig. 3. In a normal knee (Fig. 3A, B), PCCT-MRI fusion provides sharper delineation of the subchondral bone plate (arrow) while preserving the smooth cartilage contour. In a case of cartilage thinning (Fig. 3C, D), fusion enhances visualization of the subchondral bone plate (star) and facilitates more accurate assessment of cartilage loss. In a patient with a focal high-grade cartilage defect (Fig. 3E, F), the fused image reveals the residual deep cartilage layer and underlying subchondral irregularity that are less conspicuous on MRI alone.

### 3.3 Quantitative segmentation results

**Fig. 4:**
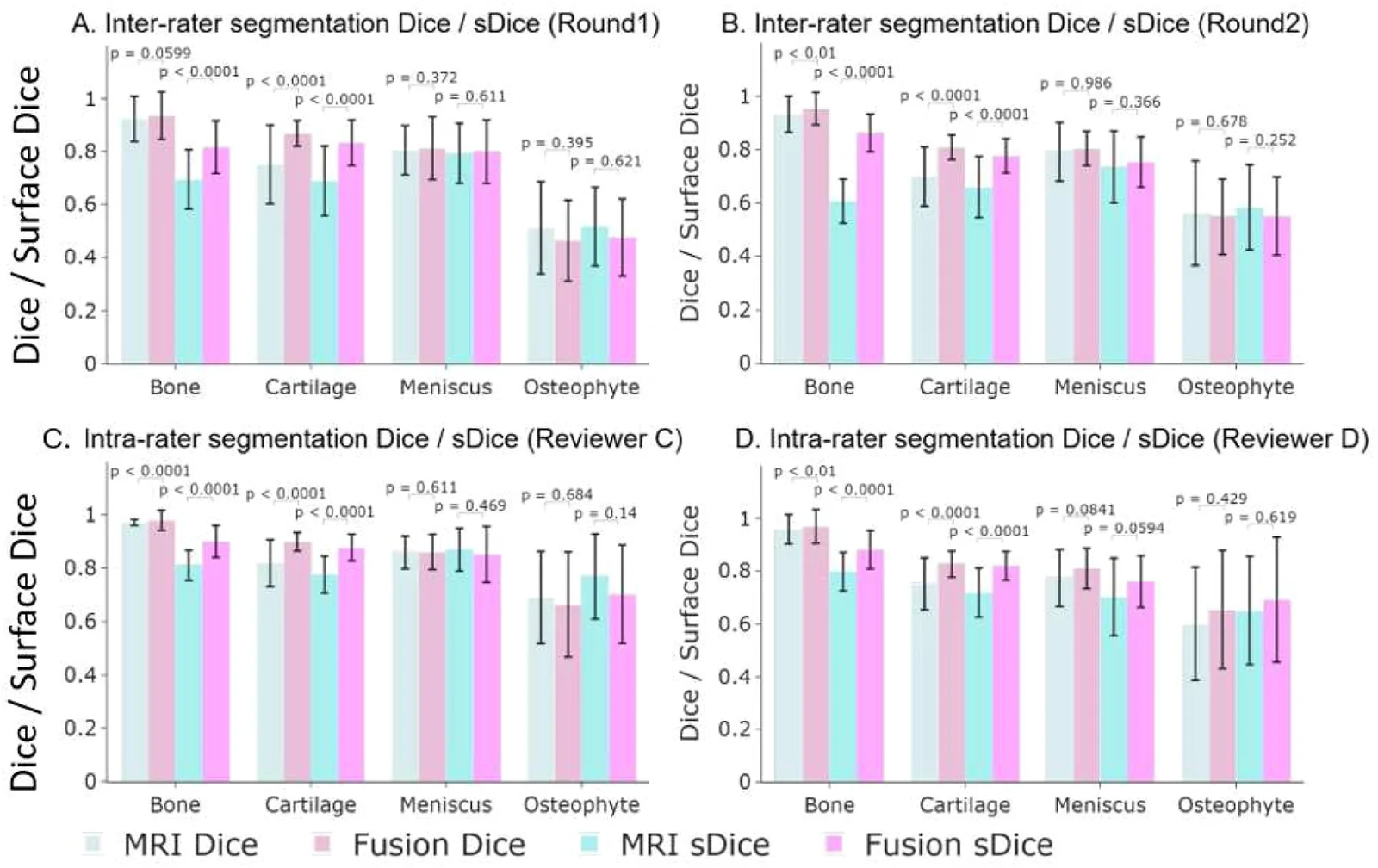
Quantitative segmentation agreement results for MRI alone and PCCT-MRI fusion. (A) Inter-rater segmentation agreement (Round 1) for bones, cartilage, meniscus, and osteophytes, reported as the Dice similarity coefficient (Dice) and surface Dice (sDice). (B) Inter-rater segmentation results for Round 2. (C-D) Intra-rater segmentation results for Reviewer C and Reviewer D, respectively, showing Dice and sDice for MRI alone and fusion images.

Fig. 4 and Table 3 summarize the inter-rater and intra-rater segmentation agreement from MRI alone and PCCT-MRI fusion images.

**Table 3:** Inter-rater and intra-rater segmentation agreement for MRI alone and PCCT-MRI fusion images. Values are presented as mean ± standard deviation (SD). Metrics include the Dice similarity coefficient (Dice) and surface Dice (sDice, τ = 1 mm). The table presents two rounds of inter-rater agreement (Round 1 and Round 2) between Reviewers C and D, as well as intra-rater agreement for each reviewer evaluated over repeated segmentation sessions.

Modality	Object	Inter-Rater (Round 1) (mean ± SD)		Inter-Rater (Round 2) (mean ± SD)		Intra-Rater (Reviewer C) (mean ± SD)		Intra-Rater (Reviewer D) (mean ± SD)	
		Dice	sDice	Dice	sDice	Dice	sDice	Dice	sDice
**MRI only**	Bones	0.92±0.09	0.70±0.11	0.93±0.07	0.61±0.08	0.97±0.01	0.81±0.06	0.96±0.06	0.80±0.07
	Cartilages	0.75±0.15	0.69±0.13	0.70±0.11	0.66±0.11	0.82±0.09	0.78±0.07	0.75±0.10	0.72±0.09
	Meniscus	0.81±0.09	0.79±0.11	0.79±0.11	0.73±0.13	0.86±0.06	0.87±0.08	0.77±0.11	0.70±0.15
	Osteophyte	0.51±0.17	0.52±0.15	0.56±0.20	0.58±0.16	0.69±0.17	0.77±0.16	0.60±0.21	0.65±0.20
**PCCT-MRI fusion**	Bones	0.94±0.09	0.82±0.10*	0.95±0.06*	0.86±0.07*	0.98±0.04*	0.90±0.06*	0.97±0.06*	0.88±0.07*
	Cartilages	0.87±0.05*	0.83±0.09*	0.81±0.05*	0.78±0.06*	0.90±0.03*	0.88±0.05*	0.83±0.05*	0.82±0.05*
	Meniscus	0.81±0.12	0.80±0.12	0.80±0.06	0.75±0.09	0.86±0.07	0.85±0.11	0.81±0.08	0.76±0.10
	Osteophyte	0.46±0.15	0.48±0.15	0.55±0.14	0.55±0.15	0.66±0.20	0.70±0.18	0.65±0.22	0.69±0.24

*Significance levels:* *p < 0.05.

#### 3.3.1 Inter-rater agreement

In the first round of inter-rater evaluation, PCCT-MRI fusion demonstrated improved segmentation consistency compared with MRI alone, particularly for bone and cartilage. For bone segmentation Dice improved from 0.92 ± 0.09 with MRI to 0.94 ± 0.09 with fusion, with sDice rising from 0.70 ± 0.11 to 0.82 ± 0.10 (*p* < 0.0001). For cartilage, the mean Dice increased from 0.75 ± 0.15 with MRI to 0.87 ± 0.05 with fusion (*p* < 0.0001), and the corresponding sDice improved from 0.69 ± 0.13 to 0.83 ± 0.09 (*p* < 0.0001). Meniscus and osteophyte segmentations showed comparable agreement between MRI and fusion, with mean Dice values remaining at 0.81 for meniscus and 0.50 for osteophytes. In the second round of inter-rater evaluation, consistent trends were observed. Fusion maintained superior agreement for bones (Dice 0.93 ± 0.07 with MRI vs 0.95 ± 0.06 with fusion, *p* < 0.01; sDice 0.61 ± 0.08 with MRI vs 0.86 ± 0.07 with fusion, *p* < 0.0001). For cartilage segmentation, fusion also demonstrated superior agreement (Dice 0.70 ± 0.11 with MRI vs 0.81 ± 0.05 with fusion; sDice 0.66 ± 0.11 with MRI vs 0.78 ± 0.06 with fusion, both *p* < 0.0001). Meniscus and osteophyte segmentation showed comparable agreement between fusion and MRI alone.

#### 3.3.2 Intra-rater agreement

Intra-rater repeatability was consistently higher for the fusion modality across both reviewers. For Reviewer C, cartilage segmentation Dice improved from 0.82 ± 0.09 with MRI to 0.90 ± 0.03 with fusion, and sDice increased from 0.78 ± 0.07 to 0.88 ± 0.05 (both *p* < 0.0001). Bone segmentation too showed significant improvement, with Dice increasing from 0.97 ± 0.01 to 0.98 ± 0.04 and sDice from 0.81 ± 0.06 to 0.90 ± 0.06 (both *p* < 0.0001). Reviewer D’s intra-rater results mirrored these findings, with cartilage Dice improving from 0.75 ± 0.10 with MRI to 0.83 ± 0.05 with fusion, and sDice from 0.72 ± 0.09 with MRI to 0.82 ± 0.05 with fusion (both *p* < 0.0001). In both reviewers’ intra-rater analyses, meniscus and osteophyte segmentations showed comparable agreement between modalities.

## 4 Discussion

Our study demonstrated that PCCT-MRI fusion images provided significantly clearer delineation of key anatomical structures, notably the cartilage-subchondral bone plate transition. These improvements were observed consistently across experienced radiologists, as evidenced by higher qualitative clarity scores and increased inter-rater agreement. Quantitatively, the fusion modality also improved both inter-rater and intra-rater segmentation agreement for critical structures such as bones and cartilages.

While the fused images showed substantial visual improvement of cartilage and osteophytes, segmentation scores were comparable for the meniscus and osteophytes, but with no substantial improvement from using the fused images. We believe that this is because the meniscus is not directly associated with, or delineated by, a high contrast structure (i.e., bone), unlike cartilage surfaces. As a result, meniscal morphology is already well visualized on MRI alone, and PCCT is unlikely to provide substantial incremental value for this specific feature. Osteophyte segmentation remained challenging despite improved visual conspicuity; osteophytes are small structures with inherently ambiguous anatomical boundaries, making segmentation variability more dependent on observer interpretation than image quality alone.

This multi-modality framework may significantly advance our understanding of OA pathophysiology in clinical research. Specifically, by simultaneously visualizing fine bony details and subtle soft-tissue abnormalities in a single fused modality, this approach offers important advantages for investigating the structural crosstalk between subchondral trabecular bone and OA-related features, including bone marrow lesions and synovitis. In particular, the dose efficiency of PCCT is an additional benefit in early OA, making repeated imaging feasible for longitudinal monitoring. Future longitudinal investigations will be needed to determine whether these detailed morphological insights can eventually be translated into targeted therapeutic strategies or prognostic biomarkers.

Despite the promising findings, this study has several limitations. First, this was an exploratory secondary imaging analysis based on a small convenience sample of participants who had completed both imaging modalities. Both the modest sample size and single-center design may limit the generalizability of our conclusions. Together, these limitations also preclude a statistically powered, stratified analysis by OA status. Future work will aim to improve the generalizability of the findings by expanding the cohort through multi-center collaborations that encompass a range of Kellgren-Lawrence grades, capturing greater variability in patient demographics, imaging protocols, and scanner platforms while enabling stratified analysis based on OA status.

Second, the distinct visual characteristics of the fused PCCT/MRI images meant that experienced radiologists could readily identify the modality being evaluated. Although we have randomized the presentation order and employed a washout period to minimize bias, the psychological impact of modality recognition cannot be entirely excluded. This lack of true blinding is a common challenge in multimodal fusion studies. Future studies with more subtle blending techniques or alternative observer models could help quantify the impact of this recognition bias.

Third, we anchored slice selection to the tibial spines to establish a reproducible baseline across subjects in segmentation analysis. However, this 2D constraint inherently overlooks the comprehensive, multi-planar structural variations of the joint outside this central compartment. Full 3D volumetric segmentation and evaluation remain essential. Future studies should explore full 3D volumetric segmentations to fully exploit the isotropic high resolution of the PCCT datasets across all anatomic regions.

Furthermore, technical factors inherent to the two modalities also pose challenges. The difference in spatial resolution is a notable issue: MRI provides an in-plane resolution of 0.42 mm but with a slice thickness of 4.4 mm, whereas PCCT achieves isotropic resolution as fine as 0.2 mm. For registration, MRI had to be designated as the fixed reference and PCCT as the moving image, since resampling MRI to the higher PCCT resolution would require extensive interpolation and could introduce artifacts or artificial structures. As a result, the superior spatial resolution of PCCT could not be fully exploited in the fused images. Strategies to mitigate the impact of resolution mismatch should be explored. For example, advanced super-resolution techniques or harmonization frameworks may help better leverage the high isotropic resolution of PCCT while preserving the integrity of MRI data.

Additionally, MRI and PCCT are based on fundamentally different tissue contrast mechanisms. MRI contrast is governed by proton density, relaxation properties, and fat-water distribution, enabling excellent delineation of soft tissues such as cartilage, meniscus, and bone marrow lesions. In contrast, PCCT contrast arises from x-ray attenuation and energy-dependent photon counting, providing high sensitivity to mineralized tissues but limited depiction of soft tissue composition. These intrinsic differences complicate direct intensity normalization and may lead to inconsistencies at tissue boundaries, particularly at the cartilage-subchondral bone plate and meniscus-cartilage interfaces. Consequently, although fusion enhances complementary information, the heterogeneity in contrast mechanisms and anatomical boundary definitions may complicate interpretation in regions where subtle soft tissue changes are clinically important. Efforts will focus on addressing the intrinsic differences in tissue contrast and boundary definition between the two modalities. Approaches such as modality-specific feature disentanglement or physics-informed fusion algorithms may enhance the consistency of signal representation at the osteochondral and meniscus-cartilage interfaces.

Another limitation of this exploratory study is that only a single fixed 50:50 PCCT/MRI blending ratio was evaluated. This weighting was empirically selected by two senior musculoskeletal radiologists to provide balanced visualization of osseous and soft-tissue structures. However, different weighting strategies may preferentially enhance specific anatomical targets. For example, greater PCCT weighting may improve delineation of cortical bone and osteophytes, whereas greater MRI weighting may better preserve soft-tissue contrast. Therefore, the impact of alternative weighting schemes, adaptive fusion strategies, and pathology-specific optimization remains to be investigated in future studies.

Future studies should also evaluate whether fusion of additional MRI sequences, including PD-weighted, T1-weighted, and multiplanar T2-FS images, will provide incremental improvements in fusion accuracy and clinical utility compared with the coronal T2-FS sequence used in this exploratory analysis. Moreover, the statistical analysis did not adjust for the nested structure of our dataset, which included bilateral knee scans from the same individuals. While acceptable for a technical validation and pilot feasibility study of this nature, this lack of adjustment for intra-subject dependency may potentially skew the precision of standard errors and associated p-values. Future studies will also go beyond our current knee-level analytical framework, employing advanced hierarchical statistical modeling, such as Generalized Estimating Equations (GEE) or Linear Mixed Models (LMMs), to account for intra-subject dependencies.

Finally, although a mutual information-based algorithm combined with deformable refinement was applied, a subset of cases still exhibited imperfect registration, especially in patients with positional misalignment and motion. To ensure analytic validity, these misregistered cases were excluded from the final evaluation. Improving registration accuracy remains a critical priority in our work. Deep learning-based registration models, or hybrid approaches that combine statistical and biomechanical priors, may help reduce position-related and motion-related misalignments and minimize failure cases. Together, these directions aim to enhance the fidelity and robustness of PCCT-MRI fusion and facilitate its broader clinical translation.

Overall, our findings support the hypothesis that PCCT-MRI fusion can enhance the diagnostic workup of knee pathology by providing more consistent, detailed, and reliable anatomical assessment than MRI alone.

## Funding

The research reported in this publication was supported by the 10.13039/100000070National Institute of Biomedical Imaging and Bioengineering under Award Number R21EB031185, the 10.13039/100000069National Institute of Arthritis and Musculoskeletal and Skin Diseases under Award Numbers R01AR081344, R01AR079442, R56AR081017, and K23AR084603.

## Declaration of competing interest

The authors declare that they have no known competing financial interests or personal relationships that could have appeared to influence the work reported in this paper.

## Appendix Supplementary materials

Image, application 1
